# The DNA methylation status of the *TERT* promoter differs between subtypes of mature B-cell lymphomas

**DOI:** 10.1038/s41408-023-00872-0

**Published:** 2023-06-26

**Authors:** Alexandra G. Kouroukli, Anja Fischer, Helene Kretzmer, Emil Chteinberg, Nivethika Rajaram, Selina Glaser, Julia Kolarova, Pavel Bashtrykov, Stephan Mathas, Hans G. Drexler, Hitoshi Ohno, Ole Ammerpohl, Albert Jeltsch, Reiner Siebert, Susanne Bens

**Affiliations:** 1 Institute of Human Genetics, Ulm University and Ulm University Medical Center, 89091 Ulm, Germany; 2 Computational Genomics, Department of Genome Regulation, Max Planck Institute for Molecular Genetics, Berlin, Germany; 3 Institute of Biochemistry and Technical Biochemistry, Department of Biochemistry, Stuttgart University, Allmandring 31, 70569 Stuttgart, Germany; 4 Charité - Universitätsmedizin Berlin, Hematology, Oncology and Tumor Immunology, corporate member of Freie Universität Berlin and Humboldt-Universität zu Berlin, Berlin, Germany; 5 Max-Delbrück-Center for Molecular Medicine in the Helmholtz Association (MDC), Group Biology of Malignant Lymphomas, Berlin, Germany; 6 Experimental and Clinical Research Center (ECRC), a cooperation between the MDC and the Charité, Berlin, Germany; 7 Department of Human and Animal Cell Lines, Leibniz-Institute DSMZ-German Collection of Microorganisms and Cell Cultures, Inhoffenstr. 7B, D-38124 Braunschweig, Germany; 8 Tenri Institute of Medical Research, Tenri Hospital, Tenri, Nara, Japan

**Keywords:** Cancer epigenetics, Hodgkin lymphoma

**Letter to the Editor**

Telomerase reverse transcriptase (TERT) plays a key role in the maintenance of chromosomal ends by adding telomeric repetitions (TTAGGG) and, thus, contributes to the preservation of chromosomal stability [1]. *TERT* expression is typically absent in normal somatic tissues. Notable exceptions are, besides spermatogonia, germinal center B-cells [2, 3]. In contrast to non-malignant tissues, telomerase activity is present and *TERT* is expressed in the majority of human cancers [4]. The regulatory mechanisms behind this feature are heterogeneous and at least partially interrelated. They include *TERT* promoter single nucleotide variants [5], *TERT* locus amplifications or rearrangements [6], *TERT* transcriptional activation via transcription factors such as ETS1 and ETS2 [7], and epigenetic changes at the *TERT* promoter and the *TERT* hypermethylated oncological region (THOR), a region upstream of the *TERT* core promoter (Fig. 1A) [8]. *TERT* promoter DNA methylation studies in solid tumors showed a correlation of *TERT* transcription with high DNA methylation of the THOR and low methylation at the *TERT* core promoter [8–11].

**Fig. 1:**
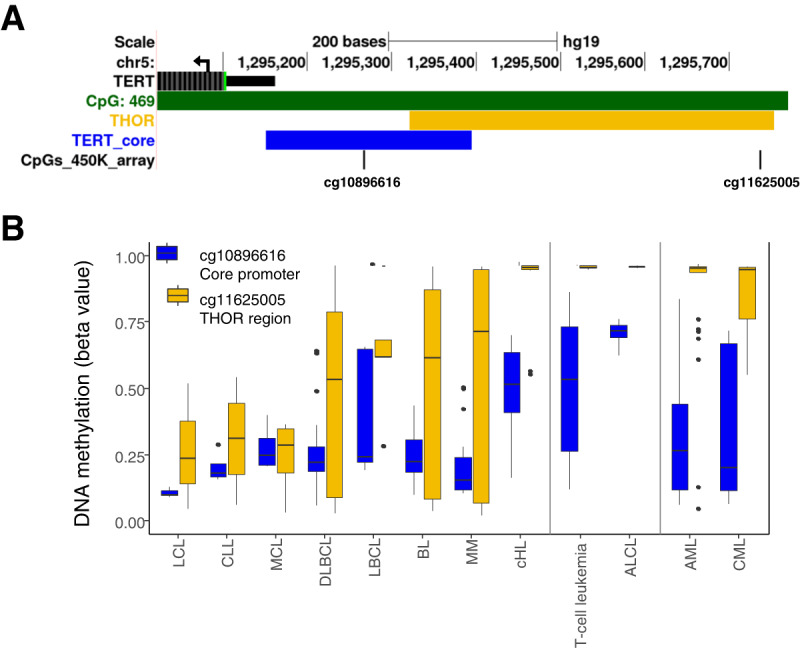
*TERT* promoter DNA methylation in hematopoietic cell lines. **A** UCSC browser view showing the *TERT* promoter region (5′ end of *TERT* gene indicated by black color with a black arrow indicating the transcription direction) and the CpG island located in this region (indicated in green color according to the CpG Islands Track from the UCSC browser). The *TERT* core promoter and the THOR region are indicated in blue and orange, respectively. CpGs present on the Illumina HumanMethylation 450k array are labeled with their TargetID. **B** DNA methylation data of 155 hematopoietic cell lines [3 lymphoblastic cell lines (LCL), 4 chronic lymphocytic leukemia (CLL), 4 mantle cell lymphoma (MCL), 33 diffuse large B-cell lymphomas (DLBCL), 5 large B-cell lymphoma (LBCL), 18 Burkitt lymphoma (BL), 16 multiple myelomas (MM), 10 classic Hodgkin lymphoma (cHL, including 2 T-lineage cell lines; HDLM-2 and L-540), 17 T-cell leukemia, 5 anaplastic large-cell lymphomas (ALCL), 29 acute myeloid leukemia (AML), 11 chronic myeloid leukemia (CML)]. DNA methylation is shown for two CpGs, one located in the *TERT* core promoter, and the other in the THOR. T-cell and myeloid malignancies are separated from the rest samples with black lines.

*TERT* expression and telomerase activity vary within and between different types of B-cell lymphomas [12]. However, despite the presence of structural variants activating TERT in B-cell lymphoma, there is little detailed knowledge available regarding the DNA methylation status at the *TERT* promoter and THOR in B-cell lymphomas. Thus, we here focused on the analysis of the *TERT* promoter and THOR DNA methylation pattern in a range of lymphomas, predominantly of B-cell origin.

For this purpose, we first analyzed available DNA methylation data generated with the Infinium® HumanMethylation450 (450 K) or MethylationEPIC (EPIC) BeadChips of 156 cell lines derived from various hematolymphoid neoplasms (137 published and 19 newly generated, Supplementary Tables 1 and 2).

We observed a profound DNA methylation heterogeneity between the two CpGs present on the array, one in the core promoter (cg10896616) and one in the THOR (cg11625005). We detected higher THOR methylation compared to core promoter methylation in BL, MM, and cHL (median beta values THOR: 0.62, 0.72, 0.96 respectively; median beta values core promoter: 0.23, 0.16, 0.52 respectively, for p-values compare Supplementary Table 3) (Fig. 1, Fig. S1). Moreover, compared to all other mature B-cell lymphoma entities, cHL cell lines showed significantly higher DNA methylation of both CpGs (Fig. 1B, Supplementary Table 3). A similar DNA methylation pattern is only observed in T-cell malignancies (Fig. 1B; median beta values core promoter: T-cell leukemia 0.54, ALCL 0.72; median beta values THOR region: T-cell leukemia 0.96, ALCL 0.96) (for *p*-values compare Supplementary Table 3). In contrast, LCLs, CLLs, and MCLs show comparably low DNA methylation in both regions (beta value < 0.5).

We further investigated 30 CpGs in the *TERT* core promoter and THOR in 43 cell lines (BS cohort: 40 lymphoma cell lines and 3 LCLs; Supplementary Table 4) using a targeted bisulfite NGS approach in order to determine if the methylation of the single CpG sites on the array represent DNA methylation at the entire *TERT* locus (Fig. 2A). The correlation between NGS and the array-based DNA methylation analysis is high with *r* = 0.79 (*p* < 0.0001; Fig. S2A).

**Fig. 2:**
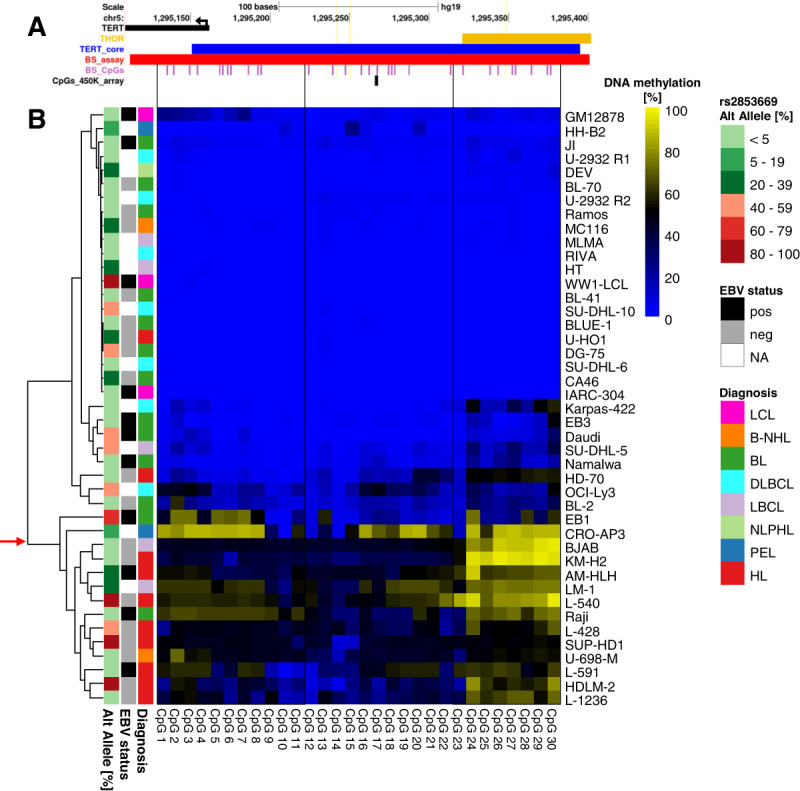
DNA methylation at CpGs flanking the *TERT* promoter in selected hematopoietic cell lines. *TERT* DNA methylation using a targeted bisulfite sequencing (BS) assay covering 30 CpGs in 40 lymphoma cell lines and three LCLs (BS Cohort). **A** UCSC browser view showing the *TERT* promoter region studied with the NGS approach. The black track corresponds to the 5′ end of *TERT* gene (black arrow indicates the transcription direction), orange track corresponds to THOR, blue track corresponds to the *TERT* core promoter as these two regions were described in Lee et al. 2019 [ref. [8]]. The red track shows the extent of the BS assay and the purple track shows the CpGs included in the BS assay. The CpG that both 450 K array and BS methods have in common can be seen in black. **B** Heatmap of relative DNA methylation [%] values in the 43 cell lines with lowest DNA methylation depicted in blue and highest in yellow. Diagnosis, EBV status and the percentage of reads with the alternative allele at the SNP position rs2853669 are annotated. Hodgkin cell lines are enriched among the samples with the highest methylation in the lower cluster indicated by a red arrow (Fisher’s exact test, *p* = 0.0006667). There are two outliers in the cHL group which display a low DNA methylation pattern in the core promoter and in THOR (U-HO1: mean DNA methylation value of 0.01 for both regions; HD-70: mean DNA methylation values of 0.23 in core and 0.48 in THOR). The heatmap is divided into 3 parts with black lines. These lines show the correspondence of CpG sites on the heatmap and the UCSC browser view.

Again, DNA methylation segregates the cell lines into two clusters with low DNA core promoter and THOR methylation in one group (most BL, DLBCL, LCLs, and most LBCLs, avg. DNA methylation < 0.05) and higher DNA methylation at both regions in the other group. Of note, the cluster displaying higher DNA methylation values is again enriched for cHL cell lines (8/14 samples are cHL cell lines in the cluster with higher DNA methylation, 57%) compared to the cluster with lower DNA methylation (2/29 cell lines are cHL cell lines, 7%, *p* < 0.001, Fisher’s exact test; Fig. 2B). Besides B-cell origin cHL, also both T-cell-origin cHL cell lines (HDML-2, L-540) were grouped in the cluster with higher DNA methylation, where the majority of cHL cell lines lie.

As cHL cell lines showed the highest DNA methylation at the *TERT* promoter and THOR, we next aimed to analyze *TERT* expression in an EBV-negative subset of 7 cHL cell lines (3 published, 4 newly generated). By RNA sequencing we detected low *TERT* expression to correlate inversely with DNA methylation values in these cell lines (Fig. S3, Supplementary Table 5).

We next expanded our analyses to 277 primary B-cell lymphomas (69 CLL/SLL, 4 MCL, 20 MZL, 30 MALT, 79 DLBCL, 25 PCNSL, 13 FL, 13 BL, 24 MM) and 110 samples from benign B-cell and progenitor populations (Supplementary Table 1). The core promoter CpG is unmethylated in the aforementioned populations which is in agreement with the lymphoma cell line data (Figs. S1, S4, and S5). RNA expression data in primary B-cell lymphomas showed a significantly higher expression of *TERT* in sporadic BL compared to DLBCL (Fig. S4B).

In order to explore a potential relationship between DNA methylation patterns and DNA sequence variants, we investigated SNPs related to ETS binding sites in the *TERT* promoter in the BS cell line cohort. We detected the alternative G allele of the SNP rs2853669, which disrupts an ETS binding site, in 20/43 cell lines by Sanger sequencing and BS, with the results of these two methods being in complete agreement. Interestingly, again the cHL subgroup stood out by carrying the alternative G allele at this SNP in 6/10 cHL cell lines being supposedly homozygous in 3/6 cHL cell lines. Considering parental allele counts, the alternative G allele was present in 9/20 alleles in cHL (allele frequency: 0.45) as compared to other B-cell lymphoma cell lines (allele frequency: 0.22) and the general population (GnomAD rs2853669 allele frequency: 0.24; Supplementary Table 6).

We next analyzed DNA methylation at CpGs 25–26 neighboring the ETS recognition site (Fig. S6) that is disrupted by the alternative G allele of rs2853669 in the BS cohort. In line with the results reported above, BL, DLBCL, most LBCL, and LCL cell lines showed low DNA methylation of the CpGs 25–26 independent of the presence of the G allele at rs2853669 (Fig. 2 and Supplementary Table 7). Apart from the cell line U-HO1 which was lowly methylated throughout the *TERT* promoter region (avg. DNA methylation value: 0.01), cHL cell lines showed high methylation at the CpGs 25–26 (Fig. 2, Supplementary Table 7). The high prevalence of the ETS site-disrupting allele along with higher DNA methylation at the neighboring CpGs in cHL cell lines prompted us to investigate the allelic DNA methylation in each of the two parts of the *TERT* promoter in cell lines carrying rs2853669. As shown in Fig. S7, cHL cell lines with DNA methylation values higher than 0.1 demonstrate differential allelic methylation in the *TERT* promoter region, with the alternative G allele showing elevated DNA methylation compared to the reference allele (Fig. S7, Supplementary Table 8).

Taken together, we observe that normal benign B-cells look similar to most of the lymphomas with respect to *TERT* promoter methylation (Fig. S4). Outliers are BL, which shows low methylation throughout the whole *TERT* promoter accompanied by activated *TERT* transcription compared to DLBCL and cHL, which are highly methylated, especially at THOR but show lower TERT expression (Figs. S1, S3, S4, and S5). Thus, the proposal that THOR DNA hypermethylation is a prerequisite for active *TERT* transcription in solid cancers [11] does not translate to BL or cHL.

Sequence variants that create ETS-family transcription factor binding motifs are reported as a mechanism for the reactivation of telomerase in solid cancers, i.e., melanoma [5]. Further studies on hepatocellular cancers show that *TERT* mutations, especially when occurring in combination with rs28553669, are associated with high *TERT* promoter methylation, leading to high *TERT* expression and poor prognosis [13]. DNA methylation data on solid tumors containing rs2853669 but lacking further *TERT* promoter mutations showed low methylation of THOR in the area surrounding this SNP [11]. Contrastingly, we here observed the presence of the alternative G allele of rs28553669 among all lymphoma subentities and no obvious correlation with *TERT* methylation or *TERT* expression. Nevertheless, there seemed to be an enrichment of the alternative G allele in cHL cell lines.

Both the locus-specific DNA methylation analyses as well as the allele-specific DNA methylation analyses presented herein do not provide an unambiguous correlation between sequence variants that interfere with ETS-family transcription factor motifs and *TERT* promoter methylation. ETS1 is known to be downregulated in cHL [14] and upregulated in many DLBCL [15]. Thus, it is intriguing to speculate that ETS family transcription factor expression levels dominate the DNA methylation status at the *TERT* promoter independent of sequence variants in lymphoid neoplasms, with high ETS levels correlating in a classic “inverse” way with low DNA methylation and vice versa.

In conclusion, as compared to the *TERT* THOR methylation patterns and their interplay with single nucleotide variants of the *TERT* promoter published in solid cancers, we did not observe similar patterns in B-cell lymphomas. Therefore, we believe that *TERT* regulation in B-cell lymphoma is a complex process governed by many different factors of both, genetic and epigenetic origin, which likely differ from solid cancers. This might be due to the fact, that *TERT* expression also physiologically occurs in mature B-cells and, thus, needs to be tightly regulated during B-cell differentiation.

## Supplementary information

Supplementary information

Supplementary tables
