# Supplementary material for: The DNA methylation status of the TERT promoter differs between subtypes of mature B-cell lymphomas
Source: Blood Cancer J. 2023 Jun 26;13(1):98. doi: 10.1038/s41408-023-00872-0 (PMC10293282; doi:10.1038/s41408-023-00872-0)
Supplement: Supplementary file 1 — Supplementary information [file 41408_2023_872_MOESM1_ESM.docx]

**SUPPLEMENTARY INFORMATION**

**SUPPLEMENTARY METHODS**

**Analysis of DNA methylation array data**

We analyzed publicly available DNA methylation array data derived from Infinium® HumanMethylation450 (450K) or MethylationEPIC (EPIC) BeadChips from a collection of benign B-cells, primary B-cell lymphomas and hematopoietic cell lines ((1-11), Supplementary Table 1) and complemented these data by newly generated DNA methylation array data for 19 additional lymphoma cell lines (Supplementary Table 2). We extracted DNA from these cell lines using the FlexiGene DNA Kit (QIAGEN, Venlo, Netherlands). A total of 500 ng of genomic DNA was bisulfite-converted using the EZ DNA Methylation kit (Zymo Research, Irvine, CA, USA) according to manufacturer’s instructions. DNA methylation was interrogated by the Infinium® HumanMethylation450 (450K) or MethylationEPIC (EPIC) BeadChips (Illumina Inc., San Diego, CA, USA) following the manufacturer’s guidelines. Raw intensity files (idat) were imported into the R programming environment (v4.1.3) using the minfi package (12). Data was normalized to controls using the preprocess Illumina function without background correction. The methylation level at each CpG site was calculated as a beta value, which varied from 0 (no methylation) to 1 (complete methylation). In order to cross-compare all studies, we converted samples run on EPIC arrays into virtual 450K arrays by filtering for CpGs present in both versions. We tested the reproducibility of public and in house data in cHL and the correlation was excellent between the datasets (r: 0.976-0.994, Figure S8). Heatmaps were generated using the pheatmap package 1.0.12 (13). Boxplots were visualized with ggplot 3.3.6.

**Cohort used for targeted bisulfite sequencing of *TERT* promoter region (BS Cohort)**

Next to the array analyses we studied the DNA methylation status at the *TERT* promoter region in more detail by targeted bisulfite sequencing in a cohort consisting of 40 lymphoma cell lines and three lymphoblastic cell lines (LCLs), hereafter termed BS Cohort. DNA was extracted from 13 Burkitt lymphoma (BL), 7 diffuse large B-cell lymphoma (DLBCL), 5 large B-cell lymphoma (LBCL), 2 primary effusion lymphoma (PEL), 2 B-cell non-Hodgkin lymphoma (B-NHL), 10 classic Hodgkin lymphoma (cHL, including also 2 T-lineage cHL lines: HDLM-2 (14) and L-540 (15)), 1 nodular lymphocyte predominant Hodgkin lymphoma (NLPHL) and 3 LCLs (n=43) using the FlexiGene DNA Kit (QIAGEN). The DNA concentration of each sample was measured using the Qubit dsDNA HS- und BR-Assay-Kits (Invitrogen, Waltham, Massachusetts, USA). All 43 DNA samples used in this study were authenticated using the GenePrint 10 System (Promega, Madison, Wisconsin, USA) according to the manufacturer’s protocol (Supplementary Table 4).

**Bisulfite treatment and targeted Bisulfite Sequencing in BS Cohort**

A total of 1000 ng of genomic DNA diluted in a final volume of 20 µl were used for bisulfite conversion and purification with the EpiTect Bisulfite Kit (QIAGEN) according to the manufacturer’s instructions. We used 2 μL of bisulfite converted and purified DNA for PCR amplification of the *TERT* promoter region (chr5:1,295,112-1,295,401, hg19, 290 bp, 30 CpGs) using the PyroMark PCR mix from the PyroMark PCR Kit (QIAGEN) and primers containing the overhang adapters from the 16S Metagenomic Sequencing Library Preparation protocol (Illumina) (Supplementary Table 9). The PCR reaction was done in a Labcycler Basic (Sensoquest, Göttingen, Germany). The conditions applied for *TERT* promoter region amplification were as follows: 15 minutes at 98 °C, seven cycles of 30 seconds at 98 °C, 30 seconds at 58-55 °C (dT: -0,5/cycle), 30 seconds at 72 °C, 38 cycles of 30 seconds at 98 °C, 30 seconds at 55 °C, 45 seconds at 72 °C and finally ten minutes at 72 °C. PCR products were purified using the AMPure XP magnetic beads following the manufacturer’s instructions (Beckman Coulter Life Sciences, Brea, California, USA). The purified PCR products underwent another PCR step in order to generate indexed libraries using the IDT for Illumina UD Indexes Plate Set A (Illumina and IDT, Coralville, Iowa, USA) and the EPM Enhanced PCR Mix (Illumina). The PCR for the generation of indexed libraries was done in a Biometra thermocycler (Jena Analytik, Jena, Germany). The PCR conditions were as follows: three minutes at 72 °C, three minutes at 98 °C, nine cycles of 20 seconds at 98 °C, 30 seconds at 60 °C, one minute at 72 °C and finally three minutes at 72 °C. The indexed libraries were purified using the same magnetic beads mentioned above and pooled libraries were generated with 100 ng from each sample. Targeted bisulfite sequencing of the *TERT* promoter region was performed on Illumina MiSeq sequencer (Illumina) using paired-end 2 × 300 cycles protocol. At least 35,478 reads were obtained for each sample after sequencing. The average bisulfite conversion efficiency rate was determined based on the read counts of methylated and unmethylated Cs outside a CpG context and was at least 97.7% in all samples (mean 99%), with only one outlier (Raji 93.92%, Supplementary Table 4).

**Evaluation of targeted DNA methylation analysis in BS Cohort**

We checked and corrected the targeted BS data for sequencing quality and adapter content using fastQC (16) and cutadapt (17). A minimum length of 100 nucleotides (nt), and a minimum quality of 25 was required to keep a read for processing. As common in Illumina sequencing, the sequencing quality values dropped towards the end of the reads, showing the expected increased accumulation of low sequencing quality scores specifically in mate 2. To ensure high data quality and reliable base calls, we trimmed 120 nt from the end of mate 2 by default as these did not meet our high-quality standards (quality scores ≥ 20). Subsequently, we aligned the reads against a gene-specific reference (further down) using BISMARK (18) with bowtie2 (19) and the non-directional protocol. Additionally, the allele-specific positions were N-masked to correct for biases in the alignment and allow for later deconvolution of allele specific methylation rates. Next, the alignment file was split based on the annotated Single Nucleotide Polymorphisms (SNPs) distinguishing the two alleles using SNPsplit (20). The split alignments were finally used for methylation calling using BISMARK’s methylation extractor function (with parameters no_overlap and comprehensive). We set the minimal number of reads for methylation rates to 500 in order to continue with further analysis. The final output of the analysis is relative DNA methylation (0% fully unmethylated and 100% completely methylated) but for direct comparison with beta values, values were converted to the range of 0-1 (0 for unmethylated and 1 for fully methylated).

**Reference sequences used for targeted DNA methylation analysis**

>hg19_dna range=chr5:1,295,112-1,295,401

NNNNNnnnnnGTGGCCGGGGCCAGGGCTTCCCACGTGCGCAGCAGGACGCAGCGCTGCCTGAAACTCGCGCCGCGAGGAGAGGGCGGGGCCGCGGAAAGGAAGGGGAGGGGCTGGGAGGGCCCGGAGGGGGCTGGGCCGGGNACCCGGGAGGGGTCGGGACGGGGCGGGGTCCGCGCGGAGGAGGCGGAGCTGGAAGGTGAAGGGGCAGGACGGGTGCCCGGGTCCCCAGTCCCTCCGCCACGTGGGNAGCGCGGTCCTGGGCGTCTGTGCCCGCGAATCCACTGGGAGCCCGGCCTGGCnnnnnNNNNN

The following positions were N-masked in order to perform allele-specific DNA methylation analysis:

| Genome | Variant name | BS assay position | Number of affected nucleotides | Observed nucleotides |
| --- | --- | --- | --- | --- |
| hg19 | rs35550267 | 142 | 1 | G/A |
| hg19 | rs2853669 | 248 | 1 | A/G |

**Gene expression analysis by RNA sequencing**

RNA was extracted from HD-70, HDLM-2, U-HO1 and SUP-HD1 cell lines using the Quick-DNA/RNA Miniprep kit according to the manufacturer’s instructions (Zymo research). The sample preparation was performed according to the "Illumina Stranded Total RNA Prep Ligation with Ribo-Zero Plus" protocol (Illumina) and sequencing was performed on the Illumina NextSeq 550 platform (Illumina). Alignment was performed using STAR (version: 2.7.10a) (21) and StringTie (v2.1.1) (22) was used to calculate gene expression values. Additionally, publically available RNA-sequencing data of KM-H2, L-1236 and L-428 deposited in GEO under GSE120330 (https://www.ncbi.nlm.nih.gov/geo/, accession date: 29.08.2022) were mined (21). For the "Illumina Stranded Total RNA Prep Ligation with Ribo-Zero Plus" protocol (Illumina, San Diego, California, USA) first, rRNA was depleted from total RNA. Then the rRNA-depleted RNA was fragmented, denatured and cDNA synthesis was performed. Subsequently, sequencing adapters were ligated to the cDNA and the products were PCR amplified. The libraries were equimolar combined and 1.8 pM of the combined libraries was loaded on the NextSeq™ 500/550 High Output Kit v2.5 (300 cycles) flow cell (Illumina). The tool StringTie (v2.1.1) (22) was used with the default parameters and a reference gtf-file of hg19 (GRCh37.p13) to calculate the gene expression of own generated alignment output (bam-files) and bam-files publically available from gene expression omnibus (GSE102330). A custom R script was then used to combine all resulted transcript per million (TPM) values per sample (R version 4.2.1) (Supplementary Table 5).

For primary lymphomas, the previously published MMML and ICGC MMML-Seq datasets were analyzed (4, 23-26). For the RNA-Seq ICGC dataset, RPKM values were filtered for the *TERT* gene, the MMML microarray dataset was filtered for the *TERT* probe (207199_at). The ICGC MMML-Seq study has been approved by the Institutional Review Board of the Medical Faculty of the University of Kiel (A150/10) and Ulm (349/11), and of the recruiting centers and includes the genomic and epigenomic study of lymphoma cell lines.

**Detection of single nucleotide variants (SNVs) found at *TERT* promoter by Sanger sequencing and BS in BS Cohort**

We initially screened the *TERT* promoter for sequence variants by Sanger sequencing and the data were further verified, in cell lines with available data, by BS. We used 100 ng of DNA from each sample in BS Cohort to perform a PCR at a 482 bp long *TERT* promoter region (chr5:1,295,043-1,295,524, hg19). We used a Labcycler Basic (Sensoquest) for the PCR reaction and the conditions were as follows: one minute at 98 °C, 30 cycles of 40 seconds at 98 °C, 30 seconds at 61 °C, 30 seconds at 72 °C, and finally five minutes at 72 °C. The PCR was performed using the AmpliTaq Gold™ 360 Mastermix (Thermo Fisher Scientific, Waltham, Massachusetts, USA) and primers designed to have universal sequences that would be used in the sequencing process (Supplementary Table 9). We purified the PCR products using the Exonuclease I (New England Biolabs, Ipswich, Massachusetts, USA) and FastAP Thermosensitive Alkaline Phosphatase (Thermo Fisher Scientific). Subsequently we performed a sequencing reaction in order to produce single-stranded DNA for sequencing. The sequencing reaction was conducted using the BigDye™ Terminator Version 3.1 Cycle Sequencing Kit (Thermo Fisher Scientific) according to the manufacturer’s protocol. Thereupon, we purified the sequencing reactions before capillary electrophoresis in order to remove the reagents that could obscure the sequencing data using the Agencourt CleanSEQ beads (Beckman Coulter Life Sciences) following the manufacturer’s instructions. Finally, we performed fluorescence-based capillary electrophoresis using the ABI Prism 3130XL Genetic Analyzer (Applied Biosystems, Waltham, Massachusetts, USA) and we evaluated the data with the Sequence Scanner Software 2 (Version 2.0, Applied Biosystems, Waltham, Massachusetts, USA). The analyzed region contains ETS-family general recognition site motives (GGAA/GGAT) between CpGs 11-12 (two sites), near CpG 21 (one site) and between CpGs 25-26 (one site). The binding site between CpGs 25-26 gets disrupted by the G alternative allele of rs2853669 (MAF ≥ 1%, GnomAD alternative G allele frequency count according to dbSNP release 155, accessed on 02.01.2023: 0.241738, reference A allele frequency count: 0.758262) while the A alternative allele of rs35550267 (MAF <1%, GnomAD alternative A allele frequency count according to dbSNP release 155, accessed on 02.01.2023: 0.000007, reference G allele frequency count: 0.999993) generates one additional ETS-family binding site between CpGs 13-14 (Figure S6). Of note, we only detected the A allele of rs35550267 in GM12878 but not in any of the lymphoma cell lines studied (Supplementary Table 6). Thus, we excluded SNP rs35550267 from further analyses.

We verified the Sanger sequencing data by screening the *TERT* promoter sequence covered by the BS assay using the Integrative Genomics Viewer (IGV) 2.5.2 software (27). To account for allelic imbalances, we used a cut-off of ≥ 5% reads from the BS for calling an allele at a known SNP position rs2853669. For the detection of putative novel sequence variants, we used a cut-off of ≥ 20% to reduce false-positive findings and limit the analysis to clonal events with rather dominant function.

By Sanger sequencing we detected alternative alleles at 4 additional variant sites (rs35226131 in BL-70, U-2932 R1/2 and U-698-M, rs35161420 in BL-70, rs1384474222 in L-428 and rs907929058 in MC116; Supplementary Table 6) As these variants affect cytosine positions (C>T and C>G) which are the targets of bisulfite conversion they could not reliably called by BS. Therefore, these variants were not considered in the allelic analyses.

**Statistical analyses**

All statistical analyses were performed using R v4.1.3. Methods used for statistical hypothesis testing are directly stated in the text, table legends or figure legends. Wilcoxon tests, Fisher’s exact test and Pearson correlation were applied with Benjamini-Hochberg correction for multiple testing where applicable. Boxplots were generated using the default ggplot2 geom_boxplot settings.

**SUPPLEMENTARY FIGURES**

**
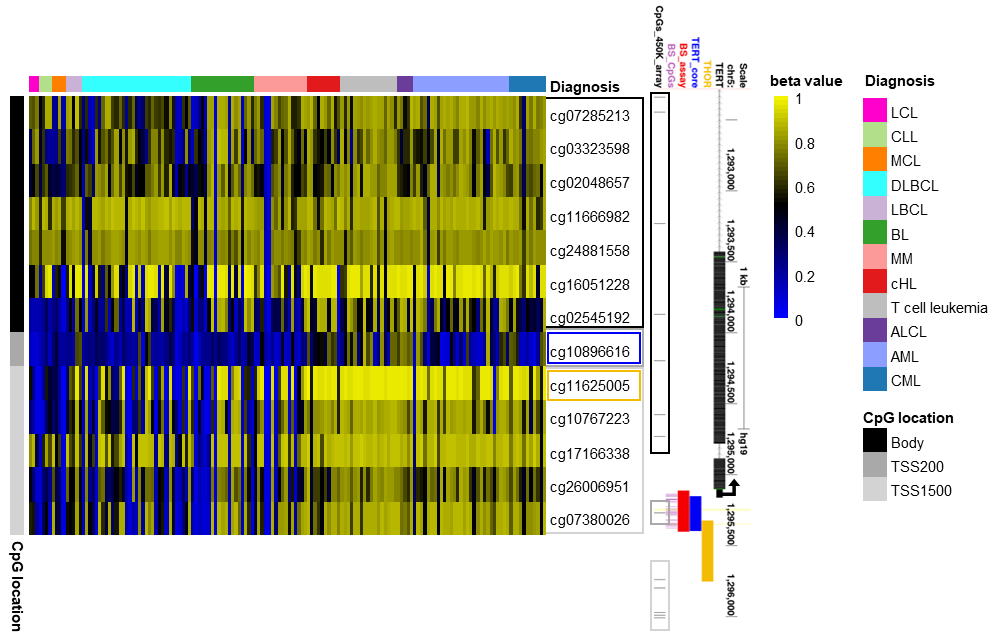
**

**Supplementary Figure 1: Array DNA methylation at CpGs flanking the *TERT* promoter in hematopoietic cell lines.** DNA methylation of 13 CpGs flanking the *TERT* core promoter (0–200 bases upstream of the transcriptional start site [TSS200]). DNA methylation data is shown for 155 cell lines with blue meaning no methylation (beta value: 0) and yellow meaning full methylation (beta value: 1). DEV cell line is not included here because of reclassification from cHL to nodular lymphocyte-predominant Hodgkin lymphoma (NLPHL) which would represent another category. On the right side of the picture there is a UCSC browser view showing the *TERT* locus including the 5’ end of *TERT* gene (black track with black arrow indicating the transcription direction), THOR (orange track), *TERT* core promoter (blue track), TERT BS assay (red track) and the 30 CpGs covered by the BS assay (shown in purple track). On the bottom of the UCSC view, the 450K CpGs can be seen indicated in black. The boxes surrounding the CpGs give information on the localization of the CpGs in respect to the *TERT* region. The black box indicates the CpGs located at the *TERT* gene body, the grey box indicates the CpG located at the TSS200 and the light grey box indicates the CpGs located 200–1500 bases upstream of the TSS (TSS1500).

**
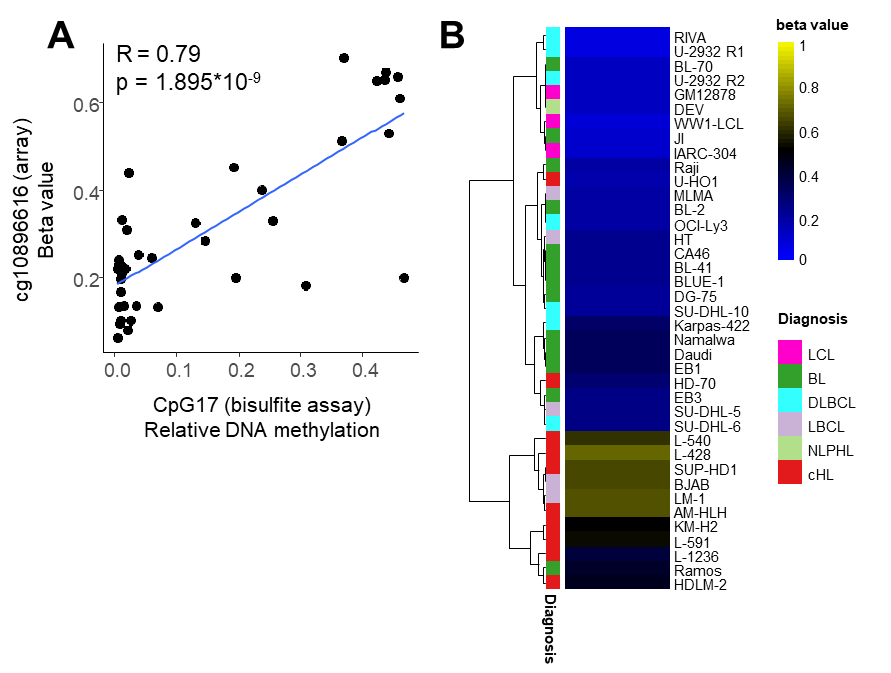
**

**Supplementary Figure 2: Correlation of *TERT* promoter DNA methylation in array data and bisulfite conversion assay. A)** Correlation of DNA methylation at the *TERT* core promoter CpG (cg10896616 in array data and CpG17 in bisulfite sequencing assay) in the 39 cell lines which are analyzed by both DNA arrays and BS. Pearson’s correlation coefficient and p value is indicated. **B)** Heatmap showing array DNA methylation at *TERT* core promoter CpG (cg10896616) in the 39 selected cell lines. Blue color means no methylation (beta value: 0) and yellow color means full methylation (beta value: 1). Hodgkin cell lines are enriched among the samples with the highest methylation in the lower cluster (Fisher’s exact test, p = 0.0001005).

**
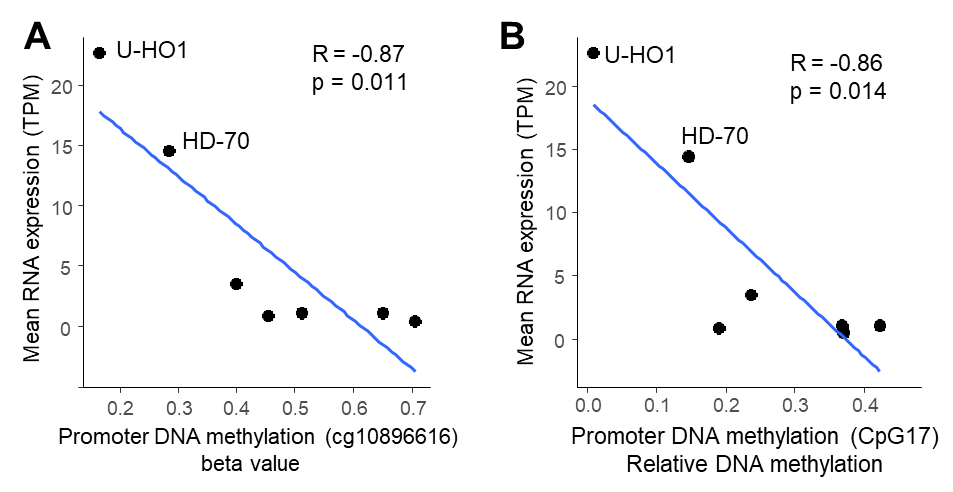
**

**Supplementary Figure 3: DNA methylation at the *TERT* promoter in Hodgkin lymphoma cell lines is inversely correlated with gene expression.** Inverse correlation of *TERT* promoter methylation with RNA expression. *TERT* RNA expression is shown as transcripts per million (TPM) counts, DNA methylation values are shown for the *TERT* promoter CpG (cg10896616) **(A)** and the CpG17 of the bisulfite assay **(B)**. For RNA sequencing samples with replicates, mean of single TPM values is shown. U-HO1 cell line demonstrates the highest *TERT* expression according to RNA sequencing (TPM value: 22.6).


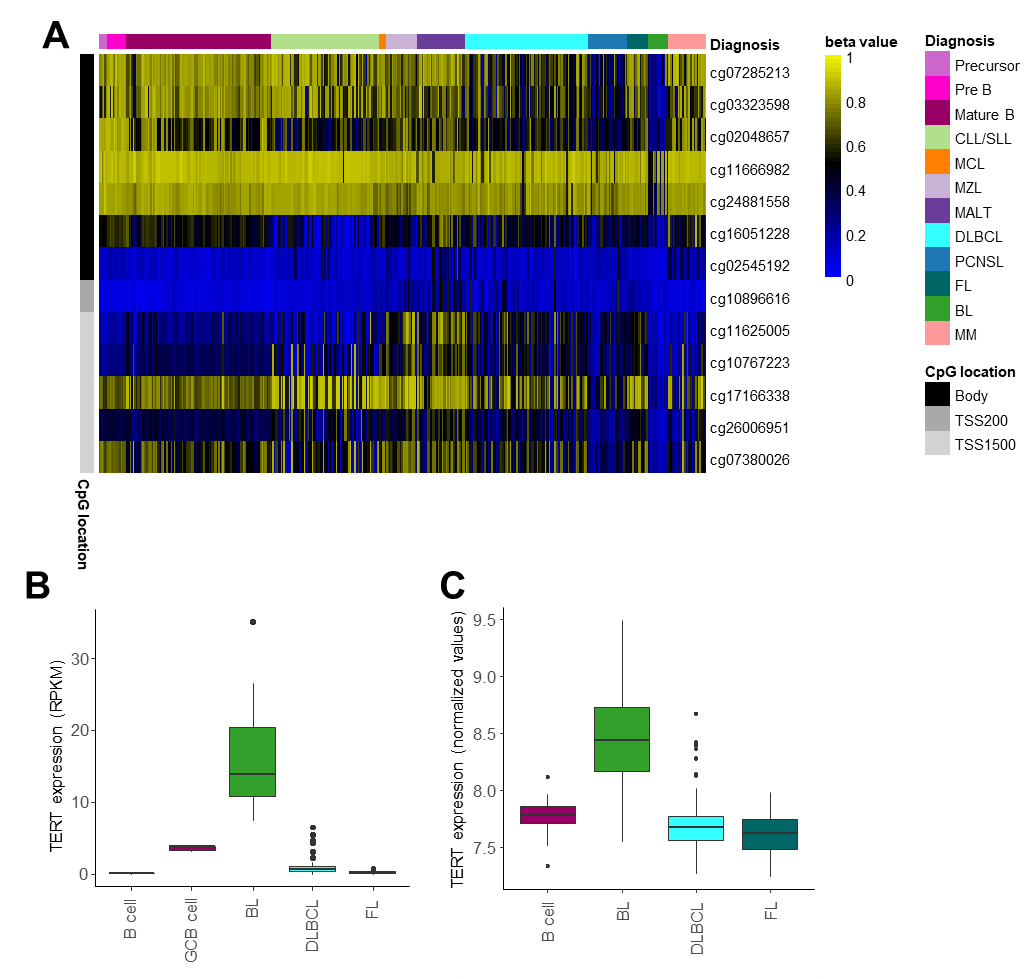


**Supplementary Figure 4: Array DNA methylation at CpGs flanking the *TERT* promoter in benign B-cells and primary B-cell lymphomas. A)** DNA methylation of 13 CpGs flanking the *TERT* core promoter (TSS200). Methylation data is shown for 387 samples [6 precursor, 12 pre B-cells, 92 mature B-cells, 69 chronic lymphocytic leukemia/small lymphocytic lymphoma (CLL/SLL), 4 mantle cell lymphoma (MCL), 20 marginal zone lymphoma (MZL), 30 mucosa-associated lymphoid tissue (MALT), 79 diffuse large B-cell lymphoma (DLBCL), 25 primary central nervous system lymphoma (PCNSL), 13 follicular lymphoma (FL), 13 Burkitt lymphoma (BL), 24 multiple myeloma (MM)]. The blue color in the heatmap indicates completely unmethylated CpGs (beta value = 0) and yellow indicates fully methylated CpGs (beta value = 1). **B)** RNA sequencing *TERT* expression data (Reads per kilobase of transcript per Million mapped reads [RPKM] values) of normal B-cells (5 B-cell, 5 GCB-cell) and primary lymphomas (21 BL, 74 DLBCL, 87 FL) from the ICGC dataset (4, 25, 26). BL vs DLBCL p adjusted = 1.7*10^-11^, BL vs FL p adjusted = 1.4*10^-11^, Wilcoxon test. **C)** Microarray *TERT* expression data of normal B-cells (40 B-cell) and primary lymphomas (71 BL, 378 DLBCL, 141 FL) from the MMML dataset (23, 24). BL vs DLBCL p adjusted = 5.9*10^-35^, BL vs FL p adjusted = 2.7*10^-29^, Wilcoxon test.

**
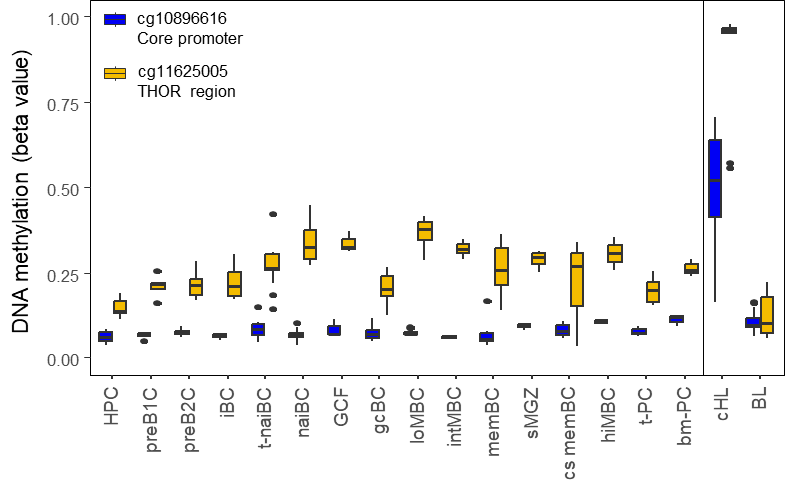
**

**Supplementary Figure 5: Differential *TERT* promoter and THOR DNA methylation in cHL and BL compared to benign B-cells.** DNA methylation array data in cHL cell lines (n = 10), BL primary lymphomas (n = 13) and benign B-cells (n = 98). DNA methylation is shown for two CpGs, one located in the *TERT* core promoter, the other in the THOR region. HPC (hematopoietic progenitor cell), preBC (pre B-cell), iBC (immature B-cell), t-naiBC (naïve B-cells from tonsils), GCF (germinal center founder cells), gcBC (germinal center B-cells), loMBC (early non class-switched memory B-cell); intMBC (non class-switched memory B-cells), memBC (memory B-cells), sMGZ (splenic marginal zone B-cells), cs memBC (class-switched memory B-cells), hiMBC (class-switched memory B-cells), t-PC (plasma cells from tonsils), bm-PC (plasma cells from bone marrow), cHL cell lines, BL cases. BL and cHL are separated with a black line from the benign cells.

**
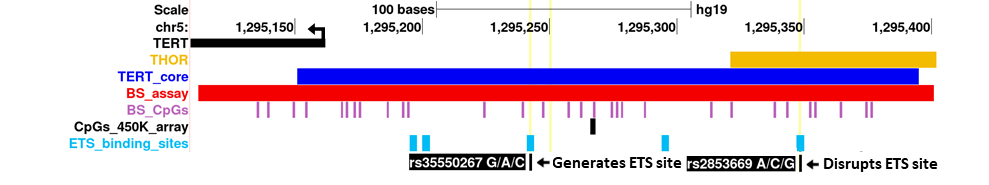
**

**Supplementary Figure 6: ETS-family general recognition sites at the *TERT* promoter region get disrupted or generated by the presence of SNPs.** UCSC browser view showing the *TERT* promoter region studied with the NGS approach (5’ end of *TERT* gene shown in black track with the black arrow indicating the transcription direction). Orange track: THOR, blue track: *TERT* core promoter as these 2 regions were described in Lee et al. 2019 (28). Red track: extend of the BS assay and purple track: CpGs included in the BS assay. The shared 450K array CpG can be seen in black and the ETS-family general recognition sites are colored with light blue. Wildtype *TERT* promoter region covered by BS assay originally has 4 ETS binding sites. Presence of the alternative A allele of rs35550267 SNP generates one additional binding site while presence of the alternative G allele of rs2853669 disrupts one already existing binding site.

**
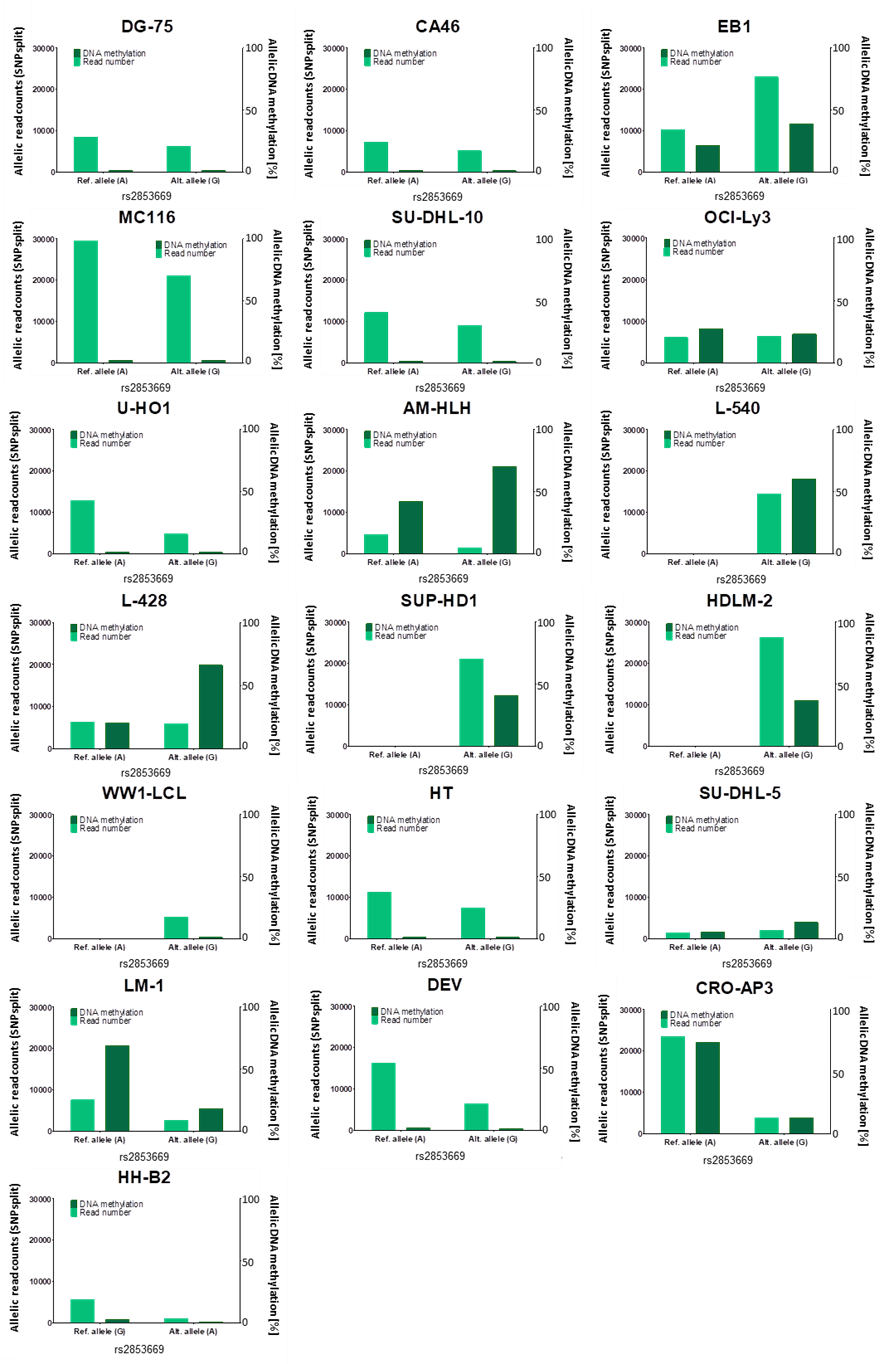
**

**Supplementary Figure 7: Overall *TERT* promoter allelic DNA methylation and allelic read counts in cell lines that contained the alternative G allele of rs2853669 SNP (chr5:1,295,349, hg19).** Allelic DNA methylation in percent (dark green) assigned on the right y axis (min: 0, max: 100). Allelic read counts (light green) according to SNPsplit report assigned on the left y axis (min: 0, max: 30,000). Due to very low number of reads, one sample (Daudi) which contains the rs2853669 SNP could not be analysed.

**
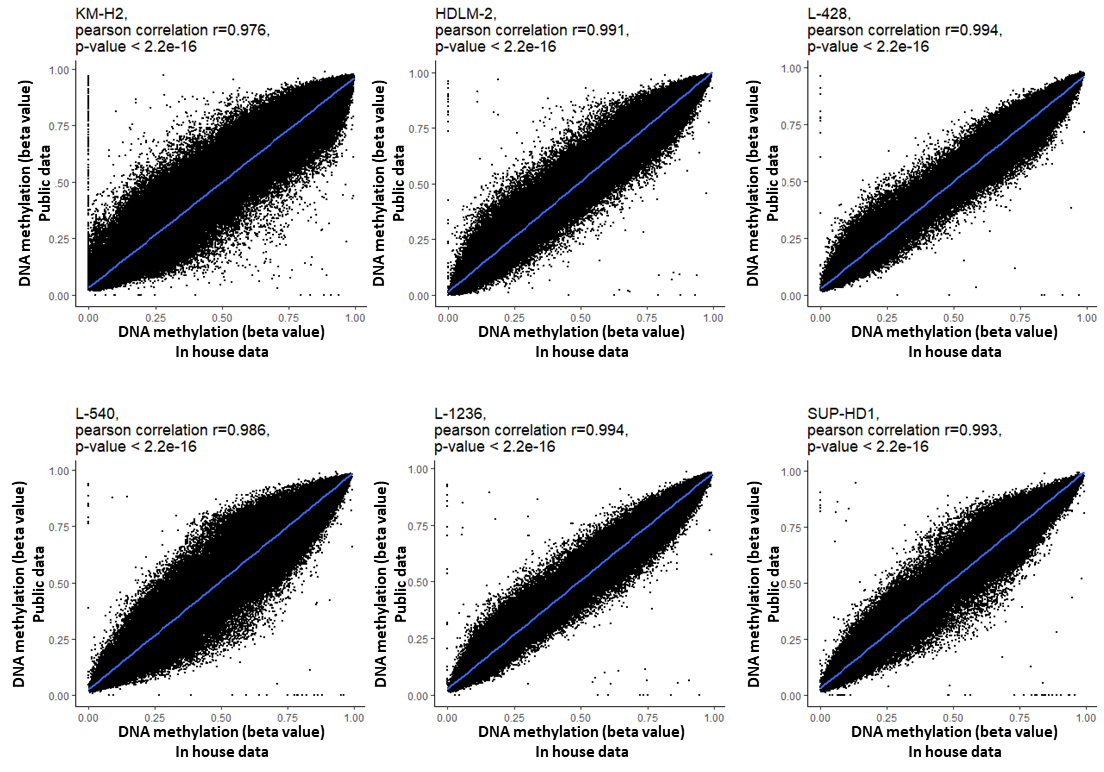
**

**Supplementary Figure 8: Correlation of publicly available and in house generated DNA methylation data in Hodgkin lymphoma cell lines.** Correlation of all 441,870 CpGs shared between Infinium® HumanMethylation450 (450K) and MethylationEPIC (EPIC) BeadChips in 6 Hodgkin cell lines. The publicly available data originated from samples run on EPIC and the in house generated data were samples run on 450K. Pearson correlation and p value are indicated.

**SUPPLEMENTARY TABLES**

**Supplementary Table 1:** Publicly available datasets used in this study.

**Supplementary Table 2**: Infinium® HumanMethylation450 (450K) beta values of CpGs flanking the *TERT* promoter in all cell lines. CLL (Chronic lymphocytic leukemia), MM (multiple myeloma), primary central nervous system lymphoma (PCNSL), follicular lymphoma (FL), Burkitt lymphoma (BL), diffuse large B-cell lymphoma (DLBCL), mucosa-associated lymphoid tissue (MALT), small lymphocytic lymphoma (SLL), marginal zone lymphoma (MZL), mantle cell lymphoma (MCL), lymphoblastic cell lines (LCL), classic Hodgkin lymphoma (cHL), acute myeloid leukemia (AML), large B-cell lymphoma (LBCL), anaplastic large-cell lymphoma (ALCL), chronic myeloid leukemia (CML).

**Supplementary Table 3:** Statistical analysis of *TERT* DNA methylation. Only comparisons with significant p values (p.adj < 0.05) are listed. Wilcoxon test was used for comparisons of DNA methylation within and between entities. P values were adjusted using the Benjamini-Hochberg (BH) method.

**Supplementary Table 4:** Table containing information about the BS cohort cell lines. Bisulfite conversion rates for the B-cell lymphomas studied. EBV and STR verification status are also shown. For the EBV status, NA means no information. Diagnosis abbreviations: Burkitt lymphoma (BL), B-cell non-Hodgkin lymphoma (B-NHL), diffuse large B-cell lymphoma (DLBCL), classic Hodgkin lymphoma (cHL), large B-cell lymphoma (LBCL), lymphoblastic cell line (LCL), nodular lymphocyte predominant Hodgkin lymphoma (NLPHL) and primary effusion lymphoma (PEL).

**Supplementary Table 5:** TPM values from RNA sequencing data for the *TERT* gene.

**Supplementary Table 6:** Read counts obtained from IGV for the SNP rs2853669 (chr5:1,295,349, hg19) which is located at the position 248 of the *TERT* BS assay (left part of the table). We used a cutoff of ≥ 5% reads from the BS for calling an allele at a known SNP position and ≥ 20% for the detection of novel variants. Each nucleotide is colored according to the IGV coloring (green for adenine [A], brown for guanine [G], blue for cytosine [C] and red for thymine [T]). The column containing the alternative allele percentage (G%) has been color-coded according to the percentage of this allele with 0% being green and 100% being red (left part of the table). Read counts obtained from the SNPsplit report for the same SNP can be seen in the middle part of the table. Same color coding has been applied for the column containing the alternative allele percentage (middle part of the table). Sanger sequencing results after screening of the *TERT* promoter region: chr5:1,295,043-1,295,524 (hg19) in BS cohort cell lines (right part of the table). The number of ETS-family general recognition sites within the region screened by BS according to the presence of SNVs can be seen on the right part of the table. SNVs that are related with C>T transitions are not detected via BS due to bisulfite treatment. All parts are separated by grey colored columns and indicated with brackets.

**Supplementary Table 7:** Converted DNA methylation values (from relative DNA methylation values with range 1-100% to a range of 0-1 for direct comparison with beta values) of the BS cohort shown in Figure 2 with blue color being completely unmethylated (DNA methylation value = 0) and yellow being fully methylated (DNA methylation value = 1). The three rows above the table show the hg19 coordinates the BS assay position and the numbering of each CpG site. The left-side columns show the name of each cell line, the EBV status (NA meaning no information available) and the diagnosis of each cell line [Burkitt lymphoma (BL), diffuse large B-cell lymphoma (DLBCL), large B-cell lymphoma (LBCL), primary effusion lymphoma (PEL), B-cell non-Hodgkin lymphoma (B-NHL), classic Hodgkin lymphoma (cHL), nodular lymphocyte predominant Hodgkin lymphoma (NLPHL) and lymphoblastic cell line (LCL)]. The two T-lineage cHL cell lines are marked in red. Average DNA methylation was calculated for the different parts of the *TERT* promoter and is shown in the right-side columns (core, THOR and overall). From the two LBCL outliers that show a higher DNA methylation pattern, LM-1 contains the rs2853669 SNP and has an average DNA methylation of 0.72 at the CpGs 25-26 which are adjacent to the disrupted ETS binding site. On the contrary, BJAB cell line from LBCL group does not contain any SNV and has an average DNA methylation of 0.82 at the CpGs 25-26.

**Supplementary Table 8**: Converted DNA methylation values (from relative DNA methylation values with range 1-100% to a range of 0-1 for direct comparison with beta values) for allele-specific DNA methylation at the *TERT* promoter region covered by the BS assay in cell lines (BS cohort) that contained SNPs. The two T-lineage cHL cell lines are marked in red. Daudi contained the rs2853669 SNP but is not shown here due to low number of reads which led to an unsuccessful allele-specific DNA methylation analysis attempt.

**Supplementary Table 9:** Primer sequences for *TERT* promoter Bisulfite and Sanger sequencing. Capital letters indicate the overhang adapters used to attach the Illumina elements such as: Flowcell binding regions for clustering, sequencing primer binding region and indexes unique for each sample. Small letters indicate the *TERT* locus-specific sequences used to amplify the region that was studied with Bisulfite sequencing. Primers were ordered from biomers.net (Ulm, Germany). For the Sanger sequencing primers small letters indicate the *TERT* locus-specific sequences and the capital letters indicate the Universal Sequences (US) used for sequencing.

**REFERENCES**

1. Kulis M, Heath S, Bibikova M, Queiros AC, Navarro A, Clot G, et al. Epigenomic analysis detects widespread gene-body DNA hypomethylation in chronic lymphocytic leukemia. Nature genetics. 2012;44(11):1236-42.

2. Lee ST, Xiao Y, Muench MO, Xiao J, Fomin ME, Wiencke JK, et al. A global DNA methylation and gene expression analysis of early human B-cell development reveals a demethylation signature and transcription factor network. Nucleic Acids Res. 2012;40(22):11339-51.

3. Agirre X, Castellano G, Pascual M, Heath S, Kulis M, Segura V, et al. Whole-epigenome analysis in multiple myeloma reveals DNA hypermethylation of B cell-specific enhancers. Genome Res. 2015;25(4):478-87.

4. Kretzmer H, Bernhart SH, Wang W, Haake A, Weniger MA, Bergmann AK, et al. DNA methylome analysis in Burkitt and follicular lymphomas identifies differentially methylated regions linked to somatic mutation and transcriptional control. Nature genetics. 2015;47(11):1316-25.

5. Kulis M, Merkel A, Heath S, Queiros AC, Schuyler RP, Castellano G, et al. Whole-genome fingerprint of the DNA methylome during human B cell differentiation. Nature genetics. 2015;47(7):746-56.

6. Puente XS, Bea S, Valdes-Mas R, Villamor N, Gutierrez-Abril J, Martin-Subero JI, et al. Non-coding recurrent mutations in chronic lymphocytic leukaemia. Nature. 2015;526(7574):519-24.

7. Iorio F, Knijnenburg TA, Vis DJ, Bignell GR, Menden MP, Schubert M, et al. A Landscape of Pharmacogenomic Interactions in Cancer. Cell. 2016;166(3):740-54.

8. Oakes CC, Seifert M, Assenov Y, Gu L, Przekopowitz M, Ruppert AS, et al. DNA methylation dynamics during B cell maturation underlie a continuum of disease phenotypes in chronic lymphocytic leukemia. Nature genetics. 2016;48(3):253-64.

9. Vogt J, Wagener R, Montesinos-Rongen M, Ammerpohl O, Paulus W, Deckert M, et al. Array-based profiling of the lymphoma cell DNA methylome does not unequivocally distinguish primary lymphomas of the central nervous system from non-CNS diffuse large B-cell lymphomas. Genes Chromosomes Cancer. 2019;58(1):66-9.

10. Dugge R, Wagener R, Mller P, Barth TFE. Genome-wide DNA methylation analysis along the progression of gastric marginal zone B-cell lymphoma of mucosa-associated lymphoid tissue (MALT) type. Br J Haematol. 2021;193(2):369-74.

11. Xia D, Leon AJ, Yan J, Silva A, Bakhtiari M, Tremblay-LeMay R, et al. DNA Methylation-Based Classification of Small B-Cell Lymphomas: A Proof-of-Principle Study. J Mol Diagn. 2021;23(12):1774-86.

12. Aryee MJ, Jaffe AE, Corrada-Bravo H, Ladd-Acosta C, Feinberg AP, Hansen KD, et al. Minfi: a flexible and comprehensive Bioconductor package for the analysis of Infinium DNA methylation microarrays. Bioinformatics. 2014;30(10):1363-9.

13. Kolde R. Package ‘pheatmap’ 2019 [Available from: <https://cran.r-project.org/web/packages/pheatmap/index.html>.

14. Drexler HG, Gignac SM, Hoffbrand AV, Leber BF, Norton J, Lok MS, et al. Characterization of Hodgkin's disease derived cell line HDLM-2. Recent Results Cancer Res. 1989;117:75-82.

15. Drexler HG, Pommerenke C, Eberth S, Nagel S. Hodgkin lymphoma cell lines: to separate the wheat from the chaff. Biol Chem. 2018;399(6):511-23.

16. Anders S. FastQC: A Quality Control Tool for High Throughput Sequence Data. 2010.

17. Martin M. Cutadapt removes adapter sequences from high-throughput sequencing reads. EMBnetjournal. 2011;17(1):10.

18. Krueger F, Andrews SR. Bismark: a flexible aligner and methylation caller for Bisulfite-Seq applications. Bioinformatics. 2011;27(11):1571-2.

19. Langmead B, Salzberg SL. Fast gapped-read alignment with Bowtie 2. Nat Methods. 2012;9(4):357-9.

20. Felix Krueger SRA. SNPsplit: Allele-specific splitting of alignments between genomes with known SNP genotypes. F1000 Reasearch. 2016;5.

21. Edginton-White B, Cauchy P, Assi SA, Hartmann S, Riggs AG, Mathas S, et al. Global long terminal repeat activation participates in establishing the unique gene expression programme of classical Hodgkin lymphoma. Leukemia. 2019;33(6):1463-74.

22. Pertea M, Pertea GM, Antonescu CM, Chang TC, Mendell JT, Salzberg SL. StringTie enables improved reconstruction of a transcriptome from RNA-seq reads. Nat Biotechnol. 2015;33(3):290-5.

23. Hummel M, Bentink S, Berger H, Klapper W, Wessendorf S, Barth TF, et al. A biologic definition of Burkitt's lymphoma from transcriptional and genomic profiling. The New England journal of medicine. 2006;354(23):2419-30.

24. Loeffler-Wirth H, Kreuz M, Hopp L, Arakelyan A, Haake A, Cogliatti SB, et al. A modular transcriptome map of mature B cell lymphomas. Genome Med. 2019;11(1):27.

25. Lopez C, Kleinheinz K, Aukema SM, Rohde M, Bernhart SH, Hubschmann D, et al. Genomic and transcriptomic changes complement each other in the pathogenesis of sporadic Burkitt lymphoma. Nat Commun. 2019;10(1):1459.

26. Hubschmann D, Kleinheinz K, Wagener R, Bernhart SH, Lopez C, Toprak UH, et al. Mutational mechanisms shaping the coding and noncoding genome of germinal center derived B-cell lymphomas. Leukemia. 2021;35(7):2002-16.

27. Robinson JT, Thorvaldsdottir H, Winckler W, Guttman M, Lander ES, Getz G, et al. Integrative genomics viewer. Nat Biotechnol. 2011;29(1):24-6.

28. Lee DD, Leao R, Komosa M, Gallo M, Zhang CH, Lipman T, et al. DNA hypermethylation within TERT promoter upregulates TERT expression in cancer. J Clin Invest. 2019;129(4):1801.
